# Systems Neuroengineering: Understanding and Interacting with the Brain

**DOI:** 10.15302/j-eng-2015078

**Published:** 2016-03-16

**Authors:** Bradley J. Edelman, Nessa Johnson, Abbas Sohrabpour, Shanbao Tong, Nitish Thakor, Bin He

**Affiliations:** 1 Department of Biomedical Engineering, University of Minnesota, Minneapolis, MN 55455, USA;; 2 School of Biomedical Engineering, Shanghai Jiao Tong University, Shanghai 200030, China;; 3 Department of Biomedical Engineering, Johns Hopkins University, Baltimore, MD 21205, USA;; 4 SINAPSE Institute, National University of Singapore, Singapore;; 5 Institute for Engineering in Medicine, University of Minnesota, Minneapolis, MN 55455, USA

**Keywords:** systems neuroengineering, neuroimaging, neural interface, neuromodulation, neurotechnology, brain-computer interface, brain-machine interface, neural stimulation

## Abstract

In this paper, we review the current state-of-the-art techniques used for understanding the inner workings of the brain at a systems level. The neural activity that governs our everyday lives involves an intricate coordination of many processes that can be attributed to a variety of brain regions. On the surface, many of these functions can appear to be controlled by specific anatomical structures; however, in reality, numerous dynamic networks within the brain contribute to its function through an interconnected web of neuronal and synaptic pathways. The brain, in its healthy or pathological state, can therefore be best understood by taking a systems-level approach. While numerous neuroengineering technologies exist, we focus here on three major thrusts in the field of systems neuroengineering: neuroimaging, neural interfacing, and neuromodulation. Neuroimaging enables us to delineate the structural and functional organization of the brain, which is key in understanding how the neural system functions in both normal and disease states. Based on such knowledge, devices can be used either to communicate with the neural system, as in neural interface systems, or to modulate brain activity, as in neuromodulation systems. The consideration of these three fields is key to the development and application of neuro-devices. Feedback-based neuro-devices require the ability to sense neural activity (via a neuroimaging modality) through a neural interface (invasive or noninvasive) and ultimately to select a set of stimulation parameters in order to alter neural function via a neuromodulation modality. Systems neuroengineering refers to the use of engineering tools and technologies to image, decode, and modulate the brain in order to comprehend its functions and to repair its dysfunction. Interactions between these fields will help to shape the future of systems neuroengineering—to develop neurotechniques for enhancing the understanding of whole-brain function and dysfunction, and the management of neurological and mental disorders.

## 1 Introduction

The brain is the single most complex component of the human body, an elaborate machine composed of interconnected parts working in harmony to control numerous conscious and subconscious functions. The neural activity that governs our everyday lives involves an intricate coordination of many processes that can be attributed to a variety of brain regions. On the surface, many of these functions can appear to be controlled by specific anatomical structures; however, in reality, numerous dynamic networks within the brain contribute to its function through an interconnected web of neuronal and synaptic pathways. The brain, in its healthy or pathological state, can therefore be best understood by taking a systems-level approach. *Systems neuroengineering* refers to the use of engineering tools and technologies to image, decode, and modulate the brain in order to comprehend its functions and to repair its dysfunction.

Several modalities have been developed to image both the organization of the brain and its neural activity. Many of these techniques, such as magnetic resonance imaging (MRI), can image the entire brain down to the millimeter level, providing high-resolution spatial information regarding the network activity associated with specific tasks or external stimuli. The dynamics of these networks can often illuminate connections or causal links among anatomical regions and provide models for how the brain processes both healthy functions and pathological ones, such as epilepsy. The imaging modalities available today can measure many types of cerebral activities, ranging from the direct neuronal output of the brain to the metabolic requirements of its function. Combining information from multiple imaging techniques can often expose the neurovascular relationships occurring within the brain and advance our understanding of its neural correlates. Imaging alone is not the only approach available to recognize the brain’s capabilities, but rather complements an assortment of other engineering techniques designed to comprehend the brain’s complex systems.

Neural interfacing technologies are of common use for decoding the neural activity related to daily bodily functions. These approaches often provide the ability to record neural activity with high temporal resolution and at multiple spatial scales, invasively or noninvasively. The brain has long been thought to function under the principles of a neural code in which specific functions elicit a stable and repeatable sequence of activity. Neural interfacing technologies allow us to detect these patterns of output across the brain and determine the corresponding behavioral or perceptual operation. One of the advantages of these techniques is the ability to detect electrophysiological responses of the brain from the cellular (single unit activity) to the system level (electroencephalography). This multi-scale opportunity can reveal detailed functionality of the brain encoded across its hierarchical structure. Motor control is one such application that has been widely explored at multiple scales, revealing physiological principles of how the brain organizes dexterous motor tasks to control prosthetic limbs using neural signals across scales, from neurons to brain rhythms.

Neuromodulation is a fast-growing field within neural engineering and offers a wide range of applications for both understanding and treating the brain. Abnormal electrical activities, such as epileptic seizures and Parkinson’s tremor, are often attributed to an array of neuropathologies and in many cases thought to be localized to specific brain regions. Whether or not this holds true, vast connections within the brain result in a cascade of atypical behavior that propagates throughout various neural networks, affecting the system as a whole. Neuromodulation technologies provide a means to alter irregular activity by stimulating the brain using a variety of electrical, optical, and sonic approaches with the goal of stabilizing the system to a healthy state. Stimulation can additionally help to uncover the mechanism of various brain processes by temporarily altering the normal function of healthy brains. Similar to neural interfacing, such perturbations can be applied both invasively and noninvasively in order to obtain and combine multi-scale information to expose the inner functionality of the brain.

It is important to note that no single technology can provide a comprehensive understanding of the brain; rather, a synergistic combination of the aforementioned techniques is required to uncover brain function. In many cases, decoding neural signals can help to identify biomarkers of a particular brain state that can be used to derive stimulation parameters needed to alter or correct the ongoing activity. Imaging results can additionally be used to inform target areas for neuromodulation such that only the desired region is stimulated. The numerous modalities available further provide the flexibility to optimize the intended alteration to best fit the subject population and intended research goal. In this review, we provide an overview of the field of systems neuroengineering with respect to neural imaging, neural interfacing, and neuromodulation and discuss the trends and challenges moving forward in these technologies at both the individual and cross-disciplinary scales.

## 2 Neuroimaging

**Figure 1.**
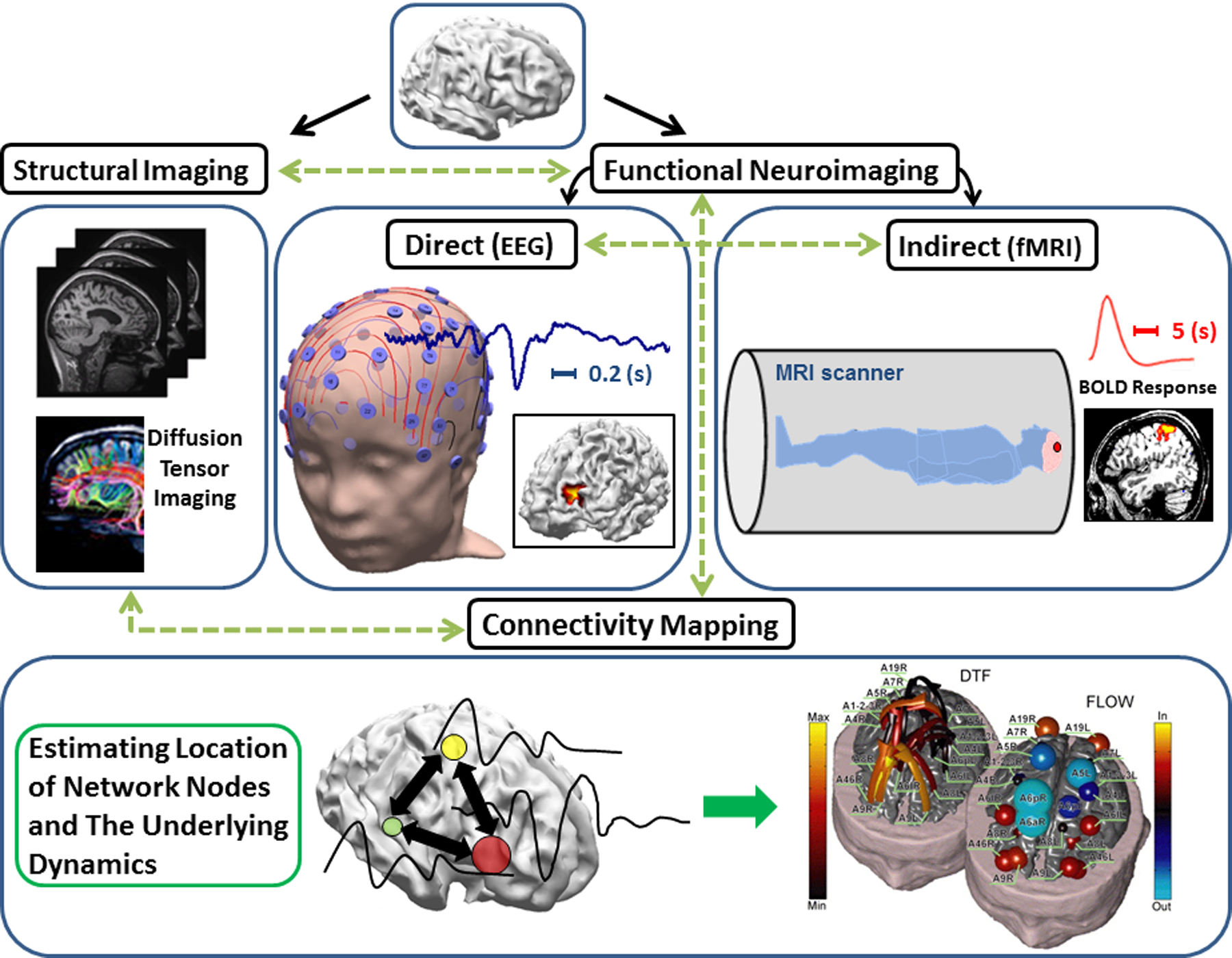
Neuroimaging at a glance: Different neuroimaging modalities interact with each other to delineate underlying brain networks (not inclusive of all modalities). Adapted from Ref. [33, 41] with permissions.

Neuroimaging represents one of the greatest achievements in modern science. Imaging can generally be separated into two different categories: structural imaging and functional imaging. While structural imaging reveals the morphology, structure, and anatomy of the brain, the aim in functional imaging is to measure perfusion rates, blood flow, electrical or magnetic signals resulting from neural excitation, and similar processes in order to delineate the functions of the system under investigation. Examples of structural imaging modalities are MRI, diffusion tensor imaging (DTI), computerized tomography (CT), ultrasound (US), fluorescence molecular imaging (FMI), and optical coherence tomography (OCT). Examples of functional imaging modalities include functional MRI (fMRI), positron emission tomography (PET), single-photon emission computed tomography (SPECT), electroencephalography (EEG), magnetoencephalography (MEG), electrocorticography (ECoG), functional near-infrared spectroscopy (fNIRS), laser speckle imaging (LSI), and photoacoustic tomography (PAT). Various structural and functional imaging modalities have come together to shed light on brain networks (Figure 1).

### 2.1 A brief introduction and survey of imaging modalities

#### 2.1.1 Structural imaging

MRI images provide high-resolution images of the brain with good contrast, making MRI a suitable choice for studying fine brain structures. CT scans, on the other hand, are fast and provide high-resolution images, but require more precaution as X-ray radiation is the basis of image acquisition in this modality [1]. In addition, its spatial resolution to image soft tissues such as the brain is limited compared with MRI. US imaging was first introduced for brain imaging in the 1990s [2], but faces attenuation challenges when transmitted through the skull. While US imaging is one of the safest imaging modalities, it has a low signal-to-noise ratio (SNR), and thus imaging deep structures of the brain remains a challenge. DTI is a modified MRI technique in which the direction of the fiber tracts in the white matter can be visualized [3]. This technique is of particular interest for high-resolution brain imaging as the wiring of different brain regions contributes to the organization of brain circuits and networks. Significant progress has been made by the Human Connectome Project (HCP) to map human brain connectivity by determining the structural and functional neural connections [4, 5]. Using DTI and tractography of white-matter fibers, the connections between different brain regions and networks are studied in a large normal population along with genetic and behavioral data [5]. Optical imaging has the advantages of superior spatial resolution (μm) as well as temporal resolution (sub-ms), though with the limitation of low penetration depth (mm). In conjunction with disease-specific fluorescent contrast agents, FMI improves the specificity of neurological disease detection at the molecular level [6]. OCT provides non-contact cross-sectional tissue imaging with micron spatial resolution and in real time, providing images of three-dimensional tissue microstructures at a penetration depth of 2 mm [7]. As a non-scanning optical technology, LSI also depicts neural vascular structure as well as angiogenesis with excellent spatiotemporal resolution [8–10].

#### 2.1.2 Functional imaging

The functional imaging of neuronal activity can be divided into direct and indirect neuroimaging [11]. Modalities such as fMRI, PET, and SPECT measure neuronal activity indirectly by detecting changes in blood flow, blood oxygenation, or glucose metabolism, while electrophysiological modalities such as EEG and MEG directly measure the electromagnetic fields generated by neuronal activity [12]. Indirect methods have low temporal resolution (in s) due to the fact that they measure slower biological processes that are modulated by neuronal activity, such as blood-flow changes, while electrophysiological recordings are more suitable to record the fast variations (in ms) of the underlying neuronal dynamics [13].

In SPECT and PET, the neuronal metabolism or blood flow (which is modulated by the neuronal activity) is measured by injecting radioisotope agents into the bloodstream [14, 15]. The minimally invasive procedure in PET and SPECT of injecting radioisotope agents carries additional risk and is a disadvantage compared to fMRI.

fMRI has been used extensively for studying brain functions and networks with high spatial resolution. However, fMRI has limited temporal resolution due to the slow hemodynamic response (the dilation of blood-vessel networks in the brain in response to increased neuronal activity), which is on the order of seconds. fMRI can detect simultaneous activation of different brain regions in response to an external stimulus (e.g., a task cue), an internal state (e.g., the interictal spikes of an epileptic patient), or during resting state, thus constituting a network of different brain regions that are time-locked with the stimuli [16]. This network can be thought of as neuronal circuits that respond, relay, or amplify specific external stimuli, or that are involved in the generation and propagation of internal stimuli. fMRI studies have grown exponentially [17] since the introduction of the modality in the early 1990s [18–20]. fMRI is currently used in a variety of basic and clinical neuroscience and psychiatric studies [21, 22]. The high spatial resolution of fMRI (mm in a 3T clinical scanner) makes it a desirable choice in localizing regions of involvement when studying neuronal networks, especially in deep brain areas that are inaccessible via other imaging modalities; however, the low temporal resolution of fMRI blinds it to the fast dynamics of neurons (which fire in the range of kHz).

EEG and MEG (E/MEG), on the other hand, can record faster activities at higher frequencies. Since E/MEG recordings are limited to the scalp, with a limited number of measurements, they have inherently limited spatial resolution compared to fMRI [23]. To determine the electrical activity of the neurons within the brain from recorded electromagnetic measurements from the surface, the electrophysiological inverse problem needs to be solved. The process of estimating the electrical activities of the neuronal ensembles from electromagnetic recordings at the surface is called electrical source imaging (ESI). Significant progress has been made in developing ESI over the past three decades, with both dipole source localization [24] and distributed source imaging approaches [25–27]. ESI techniques have been broadly used to study underlying neural circuits and pathological networks. One such application is in epilepsy, where ESI techniques can localize epileptic foci and delineate the underlying epileptogenic network [28]. Another application is in brain-machine interface, where ESI techniques can decipher the motor intent of a human subject to control a computer cursor or other external devices [29, 30].

fNIRS measures the changes in blood oxygenation when such variations are reflected in the optical properties of the brain tissue, which are measured by transmitting light beams from the scalp into the brain and measuring the resultant reflected beams [31]. fNIRS typically measures hemodynamic signals, which are indirect neuronal representations and thus have lower temporal resolution, yet fNIRS is a portable device and is more robust against motion artifacts as compared with EEG. A limitation of fNIRS is its penetration depth (due to light scattering in biological tissue), which is at most 3 cm.

LSI provides high spatiotemporal full-field blood-flow imaging with no dependence on exogenous contrast agents [32]. The brain region under investigation is illuminated by a laser light source, which ultimately forms images of the illuminated area to generate a two-dimensional blood-flow map.

**Figure 2.**
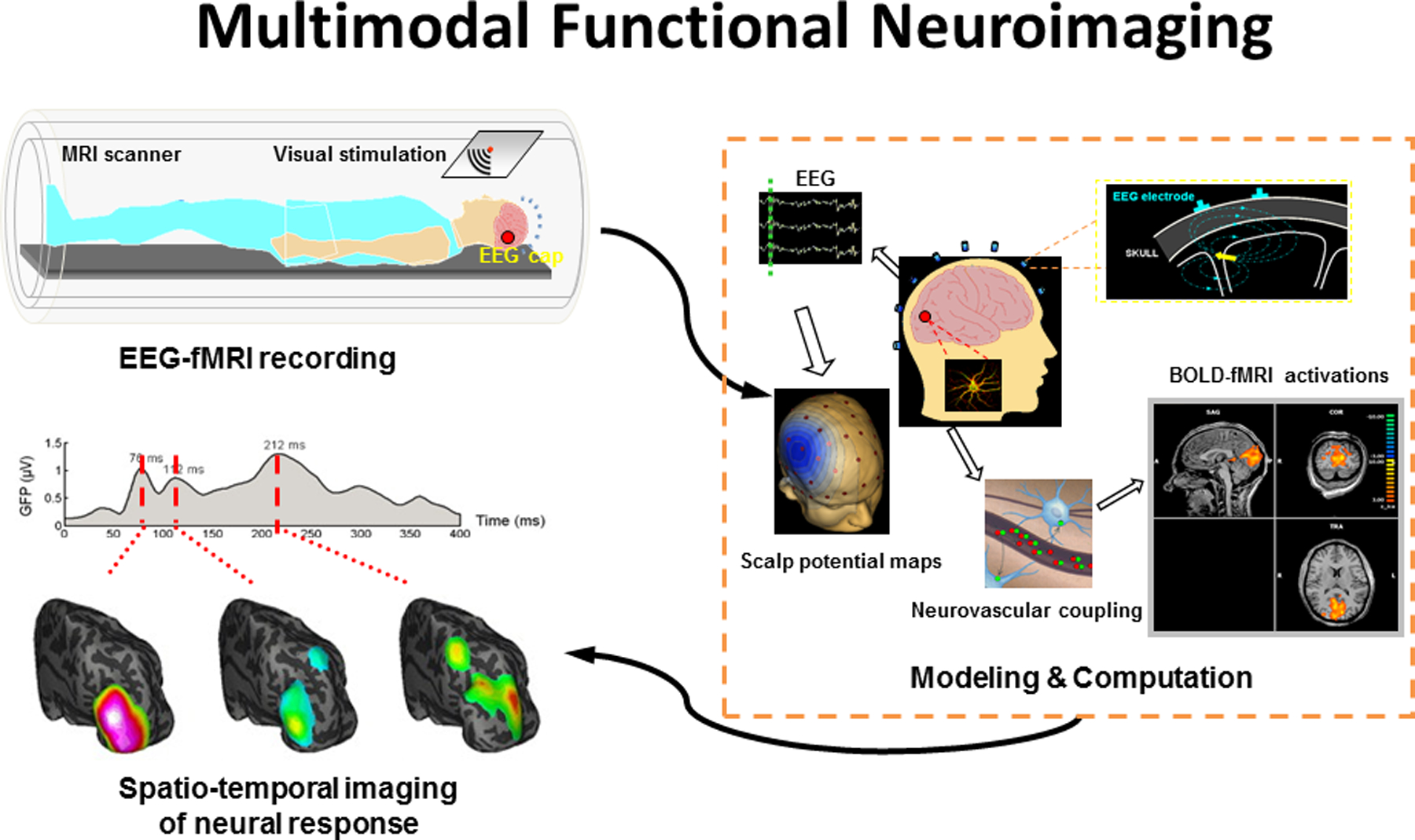
Principles of EEG-fMRI. Indirect functional neuroimaging modalities such as fMRI are related to direct electrophysiological activities via some sort of coupling (neurovascular coupling in case of fMRI). GFP: Global Field Power. Adapted from Ref. [33] with permission.

Due to the inherent limitations of individual functional imaging modalities, that is, low spatial or temporal resolution, the idea of combining different modalities to form multimodal imaging has emerged [12]. One of the most successful examples of such multimodal imaging techniques is EEG/MEG-fMRI imaging, in which the information from E/MEG and fMRI are combined together in order to elicit superior spatiotemporal resolution than is produced by each modality alone [33, 34]. Figure 2 depicts the basic principles of the EEG-fMRI technique. EEG-fMRI studies have been used in many applications such as mapping bilateral visual integration [35] and understanding epileptic networks [36].

#### 2.1.3 Networks, circuits, and connectivity

Although different regions of the brain may be involved with specialized functions, the brain is not organized as a set of independently functioning nodes. Despite the presence of functional segregation, a number of overlaps and redundancies among different regions of the brain certainly exist. These overlaps and redundancies imply that the execution of a specific task may involve many such nodes or brain regions, a concept denoted as functional integration [37]. Therefore, when considering even the simplest functions and tasks, interconnected circuits and networks are involved. In order to understand and study these networks, mathematical tools for studying connectivity and relationships among brain areas are required.

Coherence is the simplest mathematical tool for examining these associations, as it measures the similarity of different signals throughout the frequency spectrum. This tool is of particular interest because it is believed that communication between different regions of the brain is achieved through neuronal oscillations in the frequency domain as opposed to the time domain [38]. Although coherence informs us of an existing relation or link, it does not describe the direction or causality of these links [39]. In order to systematically study such causal relations within the Ganger causality framework, the directed transfer function (DTF) has been introduced [40]. DTF can simultaneously analyze and determine directed relations between different regions of interest within the brain [40, 41]. DTF is a data-driven approach that uses multivariate auto-regressive modeling of the time series under study to determine causal relationships. There are several other data-driven approaches to study connectivity, such as partial directed coherence (PDC) [42], and nonlinear methods such as mutual information [43] and generalized synchronization [44]. Linear methods are straightforward and fast to run, and have been shown to perform well in determining connectivity under most circumstances. While nonlinear methods may model the underlying connections more properly, they are generally complex and time-consuming [45, 46].

In contrast to DTF, which does not assume any underlying models for the connectivity of the network under study, methods such as structural equation modeling (SEM) [47] and dynamic causal modeling (DCM) [48] assume existing *a priori* connectivity patterns and attempt to fit the data in order to find the parameters of the model. Although SEM and DCM are successful in modeling well-studied problems with pre-existing models, the introduction of such models may bring a concerning model bias into the estimation [49]. DCM is claimed to have addressed this issue by considering alternative hypotheses (connectivity patterns) and selecting the hypothesis that best describes the data. Although this might alleviate the problem to some extent, testing multiple models is time-consuming and still requires the existence of a set of possible models to explain the underlying connectivity. DCM was originally developed for fMRI studies, but has been modified to be used with E/MEG recordings as well [46].

### 2.2 Trends, achievements, and applications

fMRI has been used extensively in studying brain networks and their underlying connectivity. Its high spatial resolution and vast field-of-view (i.e., the whole brain) makes it ideal for determining spatially distributed brain networks and circuits. fMRI studies are usually designed in a task-based or stimulus-driven fashion. In recent years however, resting state fMRI (rsfMRI) has gained popularity. In rsfMRI, the subject relaxes while the scan is in progress [50]. rsfMRI has been used to identify biomarkers in patients with various brain disorders such as autism, schizophrenia, and epilepsy.

Due to their high temporal resolution, EEG and MEG are suitable for studying brain dynamics. In a study conducted by Ding et al., seizures recorded in patients suffering from focal epilepsy were analyzed to determine the epileptogenic foci using ESI techniques; subsequently, the underlying causal networks were delineated using DTF analysis [51]. Such analyses can therefore illuminate, for example, which brain region is responsible for starting seizures or relaying seizure activity [52]. Also of great interest is the development of dynamic seizure source imaging techniques with the aid of independent component analysis (ICA) [53]; these techniques have revealed the possibility of imaging oscillatory brain activity such as a seizure.

An imaging modality will rarely have both high spatial and high temporal resolution [13]; as such, multimodal imaging has been suggested as a compromising solution to achieve higher spatial and temporal resolution than its composing sub-modalities [12]. To study networks with fast dynamics (such as in epilepsy or cognitive processes) with high spatial resolution, multimodal imaging techniques have proven useful. In epilepsy studies, fMRI activation mapping has been performed with simultaneous EEG recordings, a technique denoted as EEG-informed fMRI [36, 54]. fMRI analysis can also be used to constrain the inverse solution in ESI, usually referred to as fMRI-constrained E/MEG source imaging [33, 34], in which the spatial extent and location of fMRI activity is used to constrain the ESI solution. fMRI and E/MEG integration has its own challenges. Due to the different time scales of fMRI and E/MEG, the spatial maps provided by fMRI are relatively static and may bias the source localization results of EEG source imaging, which tend to change as quickly as the underlying E/MEG signals.

A second combined modality that has gained popularity is the PET-MRI. The results from this combination more than match the morphological data obtained from MRI and the functional images from PET. PET can provide specificity down to the molecular level, which is very important in studying metabolism and the functional activity of neurochemicals [55]. MRI and PET have been integrated in order to provide the high spatial resolution of MRI with the high specificity of PET. Still, this combined modality lacks temporal resolution, which is a desirable characteristic for studying brain circuits.

Another example of a successful combination of different modalities that circumvents the limitations of each comprising modality is PAT imaging. PAT combines optical and US technologies. Biological tissue absorbs the thermal energy emitted through a laser beam. The absorbed heat is transformed to high-frequency mechanical vibrations, which are detected by the US receivers (optical excitation and US detection). PAT is a functional imaging modality as well as a structural imaging technique [56]. For example, as the hemodynamics of the brain vessels change in response to neuronal activity, the blood oxygenation and hemoglobin concentration changes, resulting in varying absorption rates that are detected by PAT. Another multimodal imaging technique, magnetoacoustic tomography with magnetic induction (MAT-MI) [57], uses magnetic excitation to induce eddy currents that in turn produce Lorentz force, driving mechanical vibrations that are picked up by US sensors. Although MAT-MI shows promise for imaging tissue electrical properties at a millimeter spatial resolution, its application to *in vivo* brain imaging remains to be seen. Another technique, magnetic resonance electrical properties tomography (MR EPT), has shown promise for imaging functional information of brain tissue by measuring electrical properties [58] by means of transmitting and receiving radiofrequency magnetic fields.

Optical imaging has been a popular tool for neuroimaging in recent years due to its safety, high temporal resolution, and simple setup. OCT and two-photon imaging offer a superb spatial resolution at the micron level; however, other optical modalities such as fNIRS and LSI are more popular. fNIRS has shown its capability as a biomarker to measure the pathological changes of stroke, epilepsy, or affective disorders [59]. The clinical translation of fNIRS in the near future would help to evaluate and diagnose the cognitive states of the brain. LSI has been quickly developing in recent years. In principle, LSI is different from laser Doppler flowmetry; it measures the two-dimensional blood-flow speed [60], and therefore could be used to obtain the neurovascular structure pattern as well as the hemodynamic change. LSI has successfully been used to monitor cerebral blood-flow change during stroke as well as during neurovascular surgery [61]. A recent trend in optical brain imaging is to combine different optical modalities, such as LSI-OCT, in order to simultaneously obtain different neural, vascular, and hemodynamic information. Still, optical imaging methods have limited penetration depth, which is vital for noninvasive brain imaging.

### 2.3 Challenges, needs, and future trends

Brain networks are spatially distributed and interconnected and can have fast dynamics. In order to study and delineate these networks, high spatiotemporal resolution imaging modalities and techniques are needed. There is an ongoing trade-off with respect to spatial and temporal resolution in all imaging modalities, which makes it difficult for a single modality to be perfect in every sense. Achieving high resolution in both space and time proves to be a grand challenge for future neuroimaging modalities and techniques [13].

In order to increase fMRI temporal resolution, a new technique is under development called multi-band fMRI. In this approach, multiple slices of the brain are excited and subsequently imaged using various radio-frequencies that are emitted simultaneously [62]. This technique basically reduces the acquisition time of fMRI, increasing the overall temporal resolution. However, as fMRI inherently records slowly varying hemodynamic signals, the improvement in temporal resolution essentially results in higher sampling of a slow-varying signal.

For ESI techniques, new inverse algorithms based on sparse signal-processing methods have proven efficient in increasing spatial resolution [63]. Benefiting from the high temporal resolution of E/MEG, there are new ESI techniques that use redundancy in the time domain in order to provide more accurate and focal estimates of underlying neuronal activities, hence improving the spatial resolution [64, 65].

Although optical techniques and technologies enjoy high spatial and temporal resolution, their major limitation is the scattering and low penetration of light within biological tissue, which in turn decreases the field-of-view (scale) of such imaging modalities. To further overcome the low depth penetration of optical imaging techniques, multimodal imaging methodologies need to combine three-dimensional information from imaging technologies such as CT, MRI, and PET in order to compensate for limited depth penetration [66].

**Figure 3.**
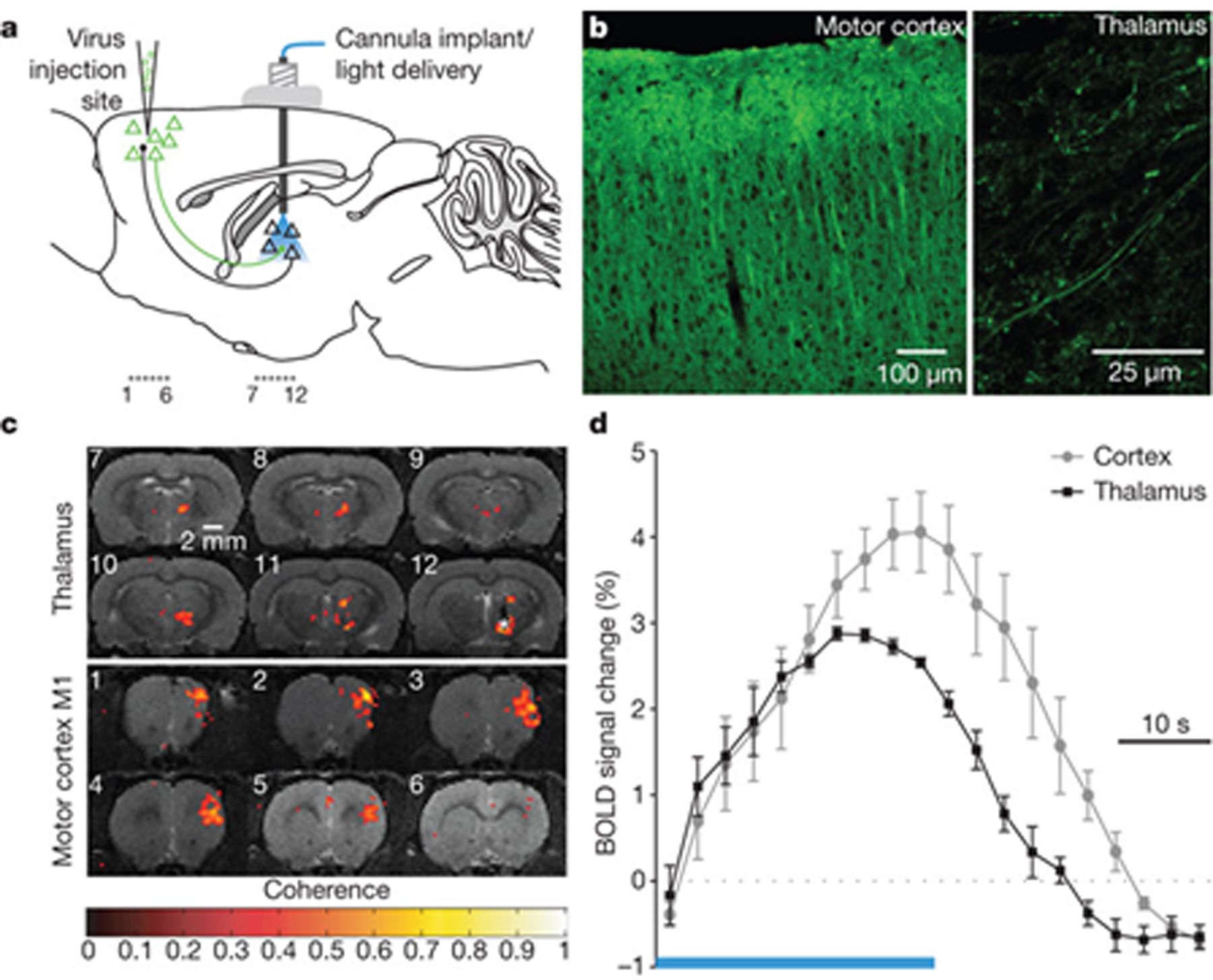
Optogenetics functional MRI (ofMRI) [69]. This technique inherits the high spatial resolution and wide field-of-view of fMRI along with the specificity of optogenetics, making it ideal for imaging selectively brain-wide large-scale networks. (a) Injecting the virus to encode specific cells to be responsive to light; (b) confirming the induction of the desired pattern; (c) the BOLD of MRI data and (d) of MRI-HRF, for BOLD signals elicited by optical stimulation. Adapted from Ref. [69] with permission.

Another important issue when studying distributed brain networks is the specificity of the circuit under study. Many brain circuits can be active simultaneously, causing interference in the signals being measured by imaging modalities. Feature extraction and component analysis are important parts of functional neuroimaging and can help to address this interference issue. A fundamentally different approach would be to stimulate and/or perturb neuronal circuits to induce activity within the desired networks or even within desired cell types, such as is performed in optogenetic approaches [67, 68]. Recently, a new technique based on this approach has been proposed called optogenetics fMRI (ofMRI), which integrates fMRI with optogenetics methods to study specific whole-brain scale networks (Figure 3) [69]. It will take some time for such techniques to be translated into clinical settings and applications due to the safety issues of injecting a viral genome into neuronal cells (a necessary step in the optogenetics method).

It is evident that the neuroimaging field is in need of technologies and techniques that are suitable for studying spatially distributed networks with fast-changing dynamics in a specific manner. This need calls for imaging modalities that have a good trade-off between spatial resolution, temporal resolution, field-of-view or scale, and specificity. Due to inherent limitations of every imaging modality and neuroimaging technique, it is highly unlikely that a single modality can achieve all of the aforementioned performance criteria. The trend in the field suggests that multimodal integration of imaging systems will be important for the grand challenges involved in neuroimaging [13].

## 3 Neural interfacing and decoding

**Figure 4.**
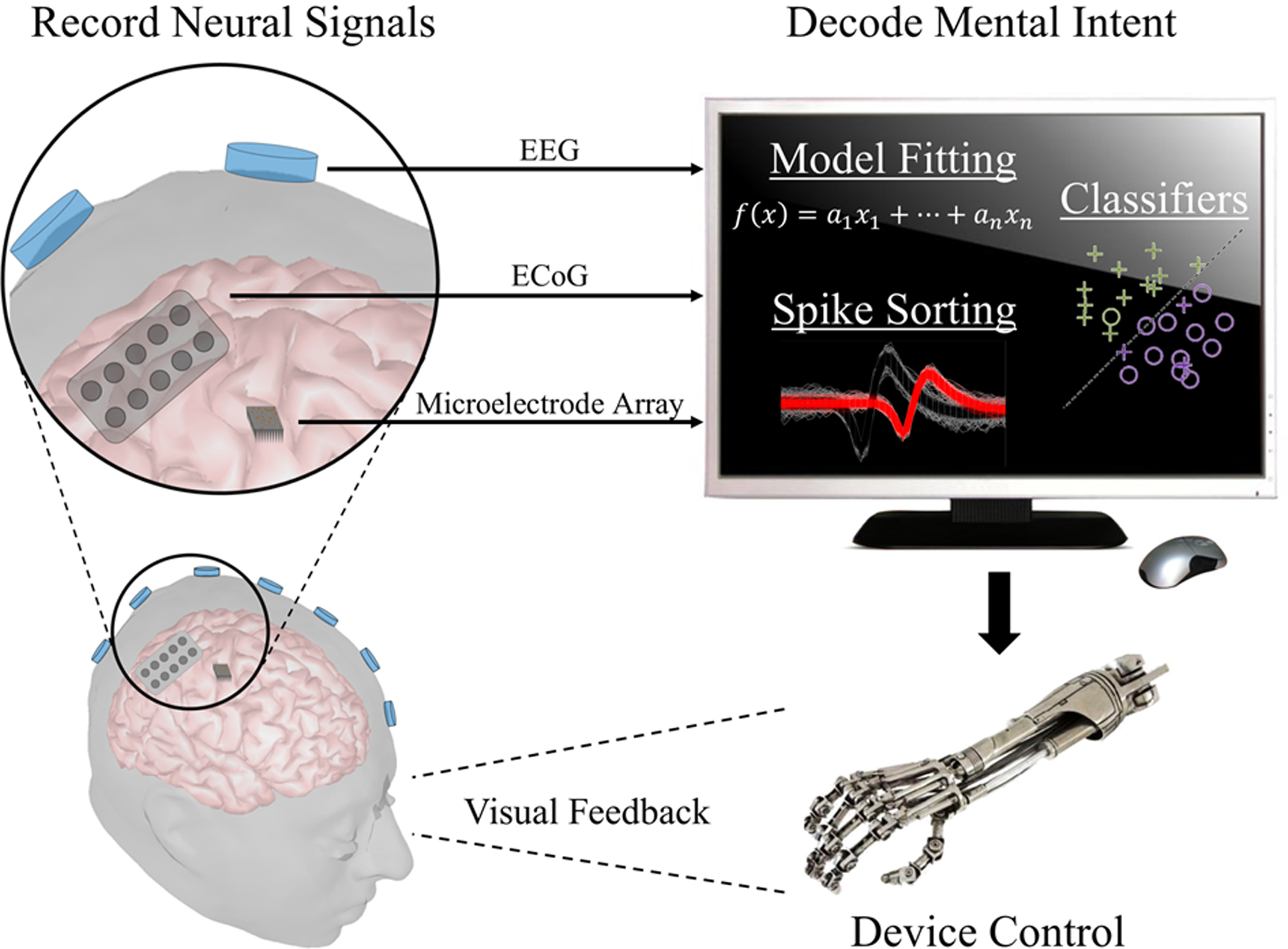
Schematic of a brain-computer/machine interface system. Signals are acquired from the brain through the use of internal or external stimuli. A computer then decodes these signals to interpret the user’s goal and translates the result into an action of the output device. Subjects can often observe such effects and modulate their brain signals to accomplish the desired task.

The concept of neural interfacing spans a variety of research areas in the field of systems neuroengineering, ranging from physical interactions between the nervous system and external devices to the interpretation and modulation of neural activity using cutting-edge technologies. In this section, we will focus on techniques that are pertinent to the latter concept in terms of direct brain communication and control. In this sense, interfacing with the brain involves the creation of bi-directional artificial pathways that resemble the physiological control systems inherent to the body. Technologies that have evolved from this ideology are known as brain-machine interfaces (BMIs) or brain-computer interfaces (BCIs). We will use BMI and BCI synonymously in this article. In general, BMIs decipher the complex signals generated by the brain for completing a specific task and provide feedback to help the user modulate or control those signals. The motivation behind these BMIs stems from the prevalence of numerous severely debilitating neuromuscular pathologies and injuries, such as amyotrophic lateral sclerosis (ALS) and spinal cord injury. In these cases, cognitive function remains intact; however, the descending motor circuitry becomes detached from the brain’s control. BMIs provide a synthetic means of conveying a user’s intentions through his or her neural activity, invasively or noninvasively, to external devices that can help the user interact with his or her environment (Figure 4).

### 3.1 A brief introduction and survey of BMI technology

#### 3.1.1 Noninvasive BMI

Noninvasive BMI approaches typically use EEG, which are measurements of the electrophysiological activity of large neuronal populations, by placing sensors on the surface of the head. Various types of signals can be decoded from these neural recordings to express the user’s mental state [70, 71].

**Figure 5.**
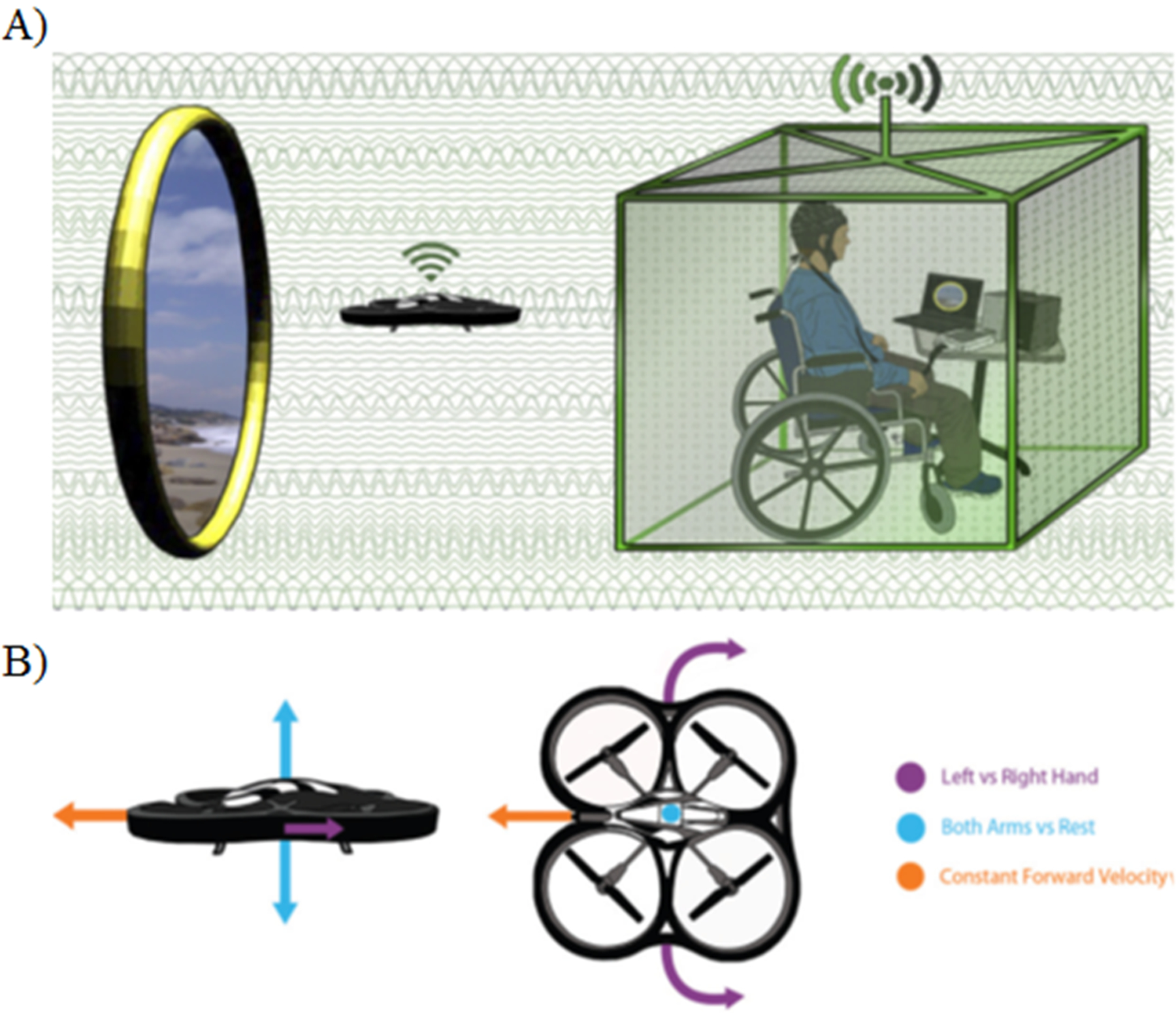
Concept diagram of using a noninvasive BMI to control a wireless quadcopter in three dimensions [77]. A camera mounted on the quadcopter allows users to view their environment. The user can then continuously navigate the quadcopter using motor imagery tasks. Adapted from Ref. [77] with permission. See Ref. [78] for a video clip demonstrating how the mind-controlled quadcopter works.

Sensorimotor rhythms (SMRs) generated in the primary motor and sensory cortices are arguably the most widely used signal for noninvasive BMI control [72, 73]. Current understanding of the behavior of SMRs for BMI applications is founded on observations of the event-related synchronization (ERS) and event-related desynchronization (ERD) phenomena that result from imagining a motor task. One-dimensional control of computer cursors was first demonstrated in the early 1990s [74] by imagining motor tasks, or by performing motor imagery (MI), of the right and left hands. These systems have since expanded to three-dimensional control using a variety of MI tasks in a virtual environment [75, 76] and have been applied to navigating quadcopters (Figure 5) [75, 77, 78] and wheelchairs [79] in real time. Despite the significant progress of EEG-based BMI, challenges remain due to the limited spatial resolution and low SNR of EEG signals. Nevertheless, various spatial filtering techniques such as the common spatial pattern algorithm [80] and EEG source imaging [29, 30], are often pursued in an attempt to overcome this signal quality issue. The virtual reality platform is another commonly used pathway to improve SMR BMI performance or explore new applications [81]. It should be noted that lengthy training protocols are often required to gain successful control of an SMR BMI; however, healthy human subjects can commonly achieve independent control of two or three degrees of freedom. Virtual reality paradigms are particularly attractive for this BMI training because they can provide an interactive environment to motivate BMI users.

BMIs utilizing event-related potentials (ERPs) were first conceptualized in the late 1980s [82] and implemented as early as the year 2000. These paradigms have been quite successful for spelling applications by exploiting the P300 component of brain ERPs. The P300 component refers to a positive scalp potential peak occurring around 300 ms after a “rare” external (or internal) stimulus is presented. These signals are commonly elicited during visual oddball paradigms where two classes of events are displayed to a subject with varying regularity. The occurrence of the less common event often provokes the endogenous P300 potential spike to occur. In these systems, a user can convey full sentences one letter at a time by attending to a desired character in an alphabetical grid composed of other undesired characters; a selection is made by sequentially highlighting rows and columns until a P300 response occurs [82]. This classic stimulus presentation grid has since been widely modified to improve the rate of communication and add system functionality. The steady-state visual evoked potential (SSVEP) is another brain signal that has been used for BMI control since the mid 1990s [83]. Subjects using these systems modulate the magnitude of frequency-specific brain oscillations by attending to external flicking stimuli. Similar to ERP BMIs, the endogenous nature of control signals requires little user training; however, a structured environment is required for stimulus presentation, and thus limits SSVEP BMIs to pre-defined setups. Robust signal detection has allowed SSVEP BMIs to achieve information transfer rates of up to 100 bits·min^−1^ [84], a significant improvement in communication over other EEG BMI signals. As such, SSVEP BMIs have been applied to a broad range of applications including virtual spellers and such external devices as hand orthoses [85]. SSVEP signals are detected by measuring the frequency-tagged power of the different stimuli using electrodes covering the occipital cortex that often do not often interfere with those used for SMR or P300 BMIs.

The distinct characteristics of the three aforementioned signals make them well suited for being combined into a single system, or hybrid BMI. Hybrid BMIs exploit the advantages of each signal type to increase BMI functionality and to overcome the disadvantages that limit each modality (for additional information on hybrid BMIs see Ref. [86]). These systems are useful if certain subjects have difficulty controlling a BMI using one type of signal. Although the fundamental limitations of each signal remain, hybrid systems can improve the use of the weaker signal by incorporating an additional signal that is more easily controlled, ultimately expanding the overall utility of noninvasive BMIs.

#### 3.1.2 Invasive BMI

In contrast to EEG-based BMIs, invasive BMIs record the activity of single neurons or small neuronal ensembles by implanting one or more arrays of microelectrodes into the cerebral cortex. It was first proposed in the early 1980s that individual neurons in the primary motor cortex were encoded with kinematic information. Small neuronal ensembles soon proved to be more sharply tuned to these signals and led to the “population vector” theory for predicting movement direction [87]. With this approach, the intended movement of a single body part can be accurately detected from multiple neurons with high degrees of freedom. Using this ideology, invasive systems have demonstrated continuous control of computer cursors using neuronal signals alone since the early 2000s [88, 89]. Such systems were quickly translated to robotic arms in reach-and-grasp paradigms in primate models [90, 91].

The ability to probe neuronal circuitry provides detailed efferent patterns related to specific kinematic movements of different body parts such that the output device can be controlled in a biomimetic fashion. Such decoders were initially constructed in primates, based on signals collected during the repeated performance of a variety of tasks. In these cases, a robust mapping is established between the task being performed and the firing rate of different cells, and can be used to decode continuous brain activity. Nevertheless, neuronal patterns used to initially construct decoders in an open-loop phase may change on even a session-to-session basis and can result in decreased performance and require re-training [89, 90]. While the results are not yet conclusive, adaptable decoders may be able to accommodate these alterations in a closed-loop fashion in order to sustain performance in BMI control over long periods of time [92]. Rather than adapting the decoder, some groups have claimed that successful adaptation can be driven in the cells being recorded from [93], by altering fixed parameters to decrease performance, only to see performance recover soon after. These cases are particularly interesting because they may provide new opportunities for studying neural plasticity or other encoding mechanisms of cells within the motor cortex.

As occurs in many cases, when the brain-electrode interface degrades over time and neuronal spikes are no longer detectable, local field potentials (LFPs) are used as an alternative source of information. While motor movements have dissimilar encoding properties for single units and LFPs, reaching and grasping kinematics can successfully be decoded in the absence of spike activity. Successful BMIs using these LFP signals have demonstrated stable biomimetic control in primates [94]. A mixed use of spikes and LFPs has also been reported for extending the longevity of invasive BMI use; as the availability of spiking activity diminishes over time, LFP signals can gradually replace the lost signal to maintain performance with a smooth transition [95]. Despite the reduction in signal quality, LFPs preserve signal spatial specificity such that usable information remains for successful biomimetic BMI control.

Invasive BMIs based on ECoG signals should also be noted here, although they are less common than those based on intracortical signals. ECoG-based BMIs utilize LFPs recorded from subdural electrode arrays placed on the surface of the brain. Compared with intracortical arrays, these electrodes provide a larger spatial sampling while sacrificing spatial specificity. ECoG was first applied to BMI technology in the mid 2000s with human epilepsy patients undergoing temporary seizure monitoring [96], and achieved successful one-dimensional cursor control. ECoG arrays bypass the skull, avoiding many of the volume conduction issues faced by EEG, and often collect signals from a large sensorimotor region. These systems are thus able to detect individual finger activity as well as kinematic information of the arm, and have shared in the success of controlling prosthetic devices [97]. Furthermore, ECoG-based BMI can be made more functional and user-friendly by building hybrid systems that augment the BMI with sensor and video capabilities [98]. Even though ECoG arrays are currently only implanted temporarily in participating patients, efforts are being made for prolonged use [99].

### 3.2 Current research and trends in BMI

#### 3.2.1 Clinical translation

In the noninvasive realm, the P300 BMI paradigm has displayed the greatest promise for use by a patient population. Applications of the P300 speller focus on patients suffering from ALS and have indicated that similar performance can be achieved in patient and healthy populations [100]. Nevertheless, disease progression can drastically affect P300 performance and systems often require customization for individual patients. For some patients, ALS has progressed to the state at which visual capabilities fade. In such cases, alternative systems based on auditory [101] or tactile [102] stimulus modalities have been implemented to maintain successful communication. BMIs using SMRs have also gained recent popularity in the rehabilitation of patients recovering from cortical stroke. Many of these studies incorporate sensory feedback by means of robotic end effectors [103, 104] to aid in the recovery process. These devices physically guide patients’ hands, often by means of passive manipulation, in response to performing MI in order to strengthen the association between imagining a motor task and performing it. Other strategies have involved a similar process using virtual reality to project an image of the hand movement being imagined onto a screen for realistic neurofeedback [105]. Such BMI training has resulted in significant functional motor recovery when compared with controls and represents a highly promising avenue for clinical use.

Several studies have proven the success of invasive BMIs in human patient populations with various forms of spinal cord injury [106, 107]. Despite limited cases, these experiments have provided paralysis patients with the ability to biomimetically control a robotic arm in reach-and-grasp paradigms [107, 108]. Specifically, in the study by Hochberg et al. [107], a female suffering from tetraplegia was able to pick up a water bottle, take a drink, and place the bottle back down on the table. Examples such as this demonstrate a key step in the clinical translation of BMIs for increasing the autonomy of debilitated patients. Other studies in primates involving functional electrical stimulation (FES) techniques have used MI signals to reanimate paralyzed limbs by stimulating the corresponding muscles involved in the task [109, 110]. When eventually translated to human populations, these systems have the possibility to fully replace the damaged spinal cord and provide patients with an enabling technology for living a more independent lifestyle [111].

#### 3.2.2 Somatosensory feedback

Sensory feedback by means of vibrotactile stimulation [112] is currently the primary alternative to visual feedback in noninvasive BMIs. The idea behind this approach is to free the visual pathway in order to attend to other tasks useful for BMI control, such as spatial navigation and obstacle avoidance. This haptic approach has been proven to be a suitable replacement for visual feedback by closing the sensorimotor feedback circuit, and has allowed more realistic navigation-based tasks to be completed. Other approaches have experimented with proprioceptive feedback [113] with promising results; however, challenges remain for isolating any one aspect of somatosensation from others in a noninvasive setting.

**Figure 6.**
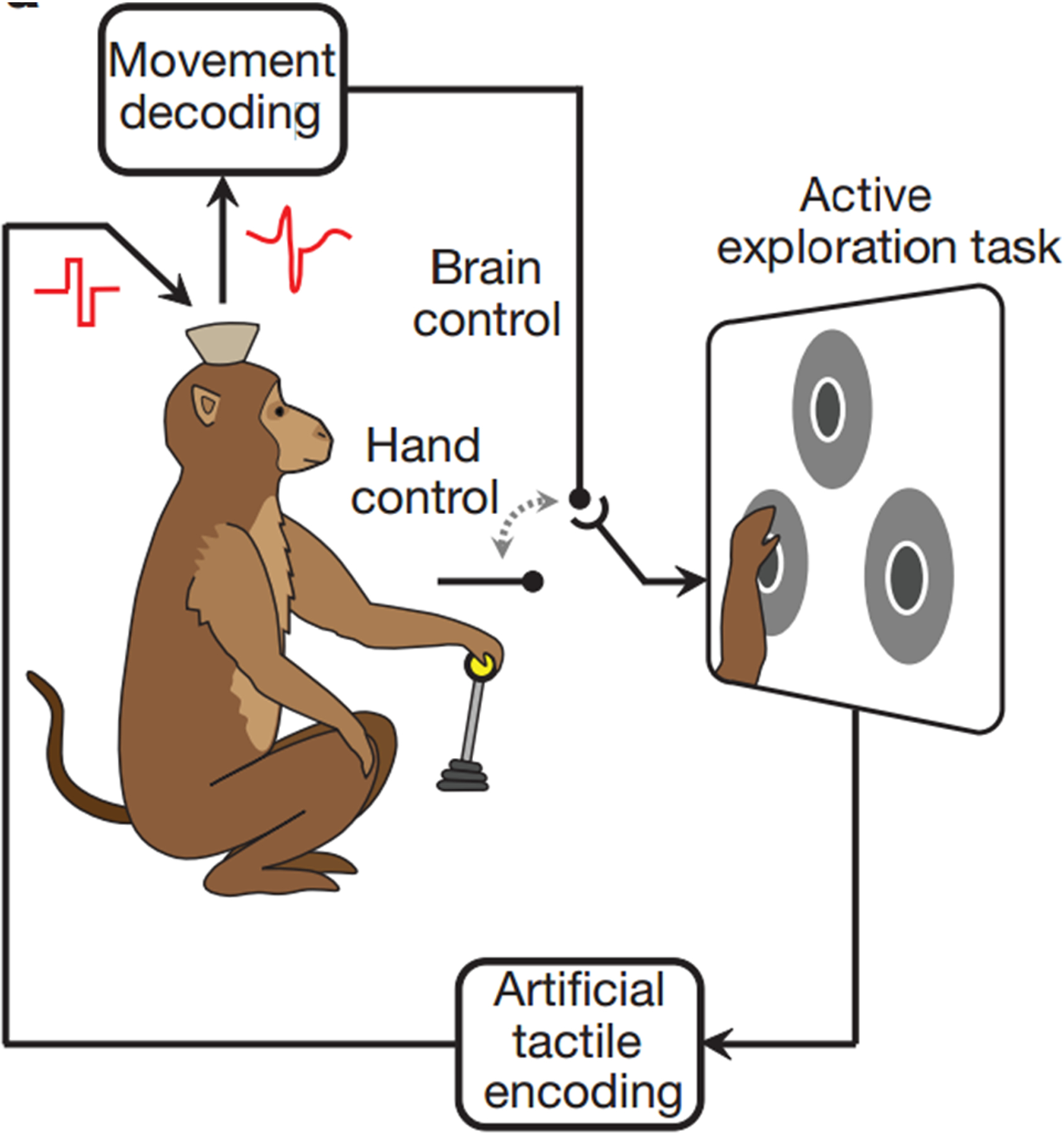
Schematic of a bi-directional BMI [116]. Motor intent is decoded from signals collected in the primary motor cortex. As the controlled device interacts with the environment, sensory cues are translated into pulse trains and used to stimulate the primary sensory cortex. In this approach, both efferent and afferent signals contribute to BMI control. Adapted from Ref. [116] with permission.

In the 1990s, micro-stimulation of the primary sensory cortex in primates was shown to elicit cognitive sensory perception of tactile stimuli similar to that of mechanical stimuli applied at the periphery [114]; however, this technique was not applied to BMIs until recently [115]. As depicted in Figure 6, micro-stimulation of the primary somatosensory cortex of a primate, while the primate controls a BMI, can provide the sensation of touch [115, 116]. Stimulating the primary somatosensory cortex using its somatotopy can provoke the basic perception of touch; however, this is only a small piece of the information required in order to mimic the neuronal activity that occurs during environmental interactions. In general, artificially inducing sensory percepts requires stimulation in order to address the different sensory features naturally occurring within the nervous system.

### 3.3 Challenges and future directions

The vast differences between noninvasive and invasive BMIs result in unique challenges in the progression of these systems toward independent and everyday use. The obvious benefits of noninvasive BMIs include the safety of recording signals from the scalp and the broad applicability of such a technique to patients or even to the general population. At the same time, signal quality and reliability issues represent challenges to realizing high-dimensional control in noninvasive BMIs. Nevertheless, advancements in signal acquisition hardware and machine-learning algorithms can further improve the usability of noninvasive systems. For example, recent progress in electrode design now offers the ability to measure electrical signals with epidermal electrodes [117]. Solving the ESI problem has also suggested a significantly improved performance of classifying natural MI tasks in an offline setting [29, 30]. Furthermore, commercialization of noninvasive BMI technology has caused a surge of new devices that allow for dry and wireless recordings that can achieve increasingly lower skin impedances while allowing users to move about their environment.

Another challenge for noninvasive BMIs is to shorten the training required to master BMI skills, despite various attempts to find biomarkers for predicting BMI performance [118]. Innovative training paradigms, such as meditation and yoga-based practices [119], have been shown to help users gain better control of a BMI; however, the mechanism of action must be better understood in order to develop optimal strategies. Other hybrid paradigms featuring SMRs and tactile stimulation have also attempted to address the illiteracy problem with promising results [120]. In general, advances made to noninvasive BMIs must act toward the single goal of driving these systems toward easy usage in the general population.

Invasive BMI technology faces its own challenges in moving forward, with safety being of the utmost concern. A head post often accompanies the implanted electrode array in order to guide wiring from the internal arrays to the external processing equipment. This device exposes the brain to the outside environment and poses a significant risk of infection, as was exemplified by the well-known BrainGate experiments [107, 121] where the head posts had to be removed from participants due to safety issues. Wireless telemetry-based devices may increase the longevity and safety of these systems; however, the need for the invasive implantation of electrodes remains an unavoidable limiting factor for its broad application to the general population.

While the decoding of efferent kinematic signals has been widely explored, the fine motor control associated with many daily tasks will require these systems to further accommodate increasing degrees of freedom. Successful separation of up to 20 dexterous manipulations of the hand has been achieved offline [122, 123], but will need to be successfully translated into online control in the future [108]. Furthermore, it was recently discovered that MI signals could successfully be extracted from the human posterior parietal cortex [124], indicating that additional neural correlates of motor control have yet to be discovered. Similarly, only the surface of somatosensation restoration has been explored, and much information is still needed regarding the encoding properties of the brain for truly biomimetic BMI control.

## 4 Neuromodulation

The field of neuromodulation—interacting with and modulating the nervous system through stimulation—has progressed from invasive brain mapping via electrical stimulation to include implantable stimulation technology and noninvasive approaches. Neuromodulation has emerged as a major area of research in the field of neuroengineering in recent years, allowing for interaction with the nervous system through a variety of technologies. Neural stimulation technologies can excite, inhibit, or disrupt brain network dynamics in a controlled fashion, depending on the stimulation parameters and application. Such technologies often offer higher specificity than medication as well as reversibility, as compared with surgical alternatives. Although most neuromodulation technologies employ electrical currents to elicit stimulation, other approaches using magnetic induction or photonic stimulation are emerging. Neuromodulation systems span multiple scales and can generally be classified into two categories, invasive and noninvasive, based on the level at which the system interacts with the nervous system.

### 4.1 A brief introduction and survey of neuromodulation technology

#### 4.1.1 Invasive neuromodulation

**Figure 7.**
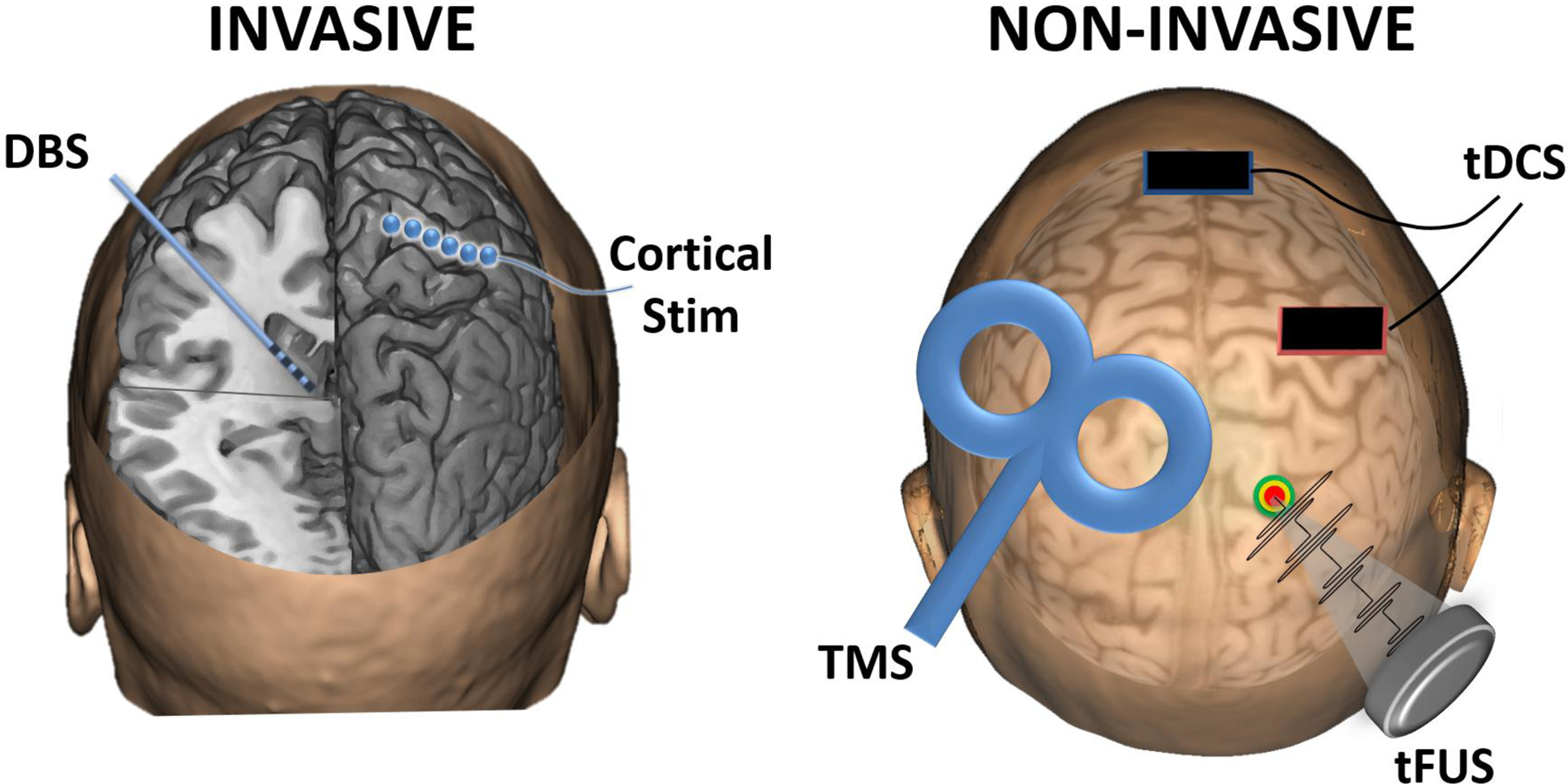
A summary of invasive and noninvasive neuromodulation technologies. Invasive techniques include DBS, in which a lead is implanted into a deep brain structure, and cortical stimulation, in which electrodes are placed on the brain surface. Noninvasive techniques include transcranial magnetic stimulation (TMS), typically applied with a figure-eight coil, transcranial direct-current stimulation (tDCS) via scalp sponge electrodes, or transcranial focused ultrasound stimulation (tFUS) using pulsed ultrasound from a transducer on the scalp.

Although the field of neuromodulation was initially based on direct cortical stimulation during surgical procedures, the invasive neuromodulation field has expanded to include a variety of additional therapeutic approaches, such as deep brain stimulation (DBS), and intracranial cortical stimulation, as shown in Figure 7. The primary advantage of invasive neuromodulation approaches is the direct interaction with neural tissue, offering higher specificity; however, such direct contact also carries the risk of inflammation, gliosis, and cell death [125].

**Figure 8.**
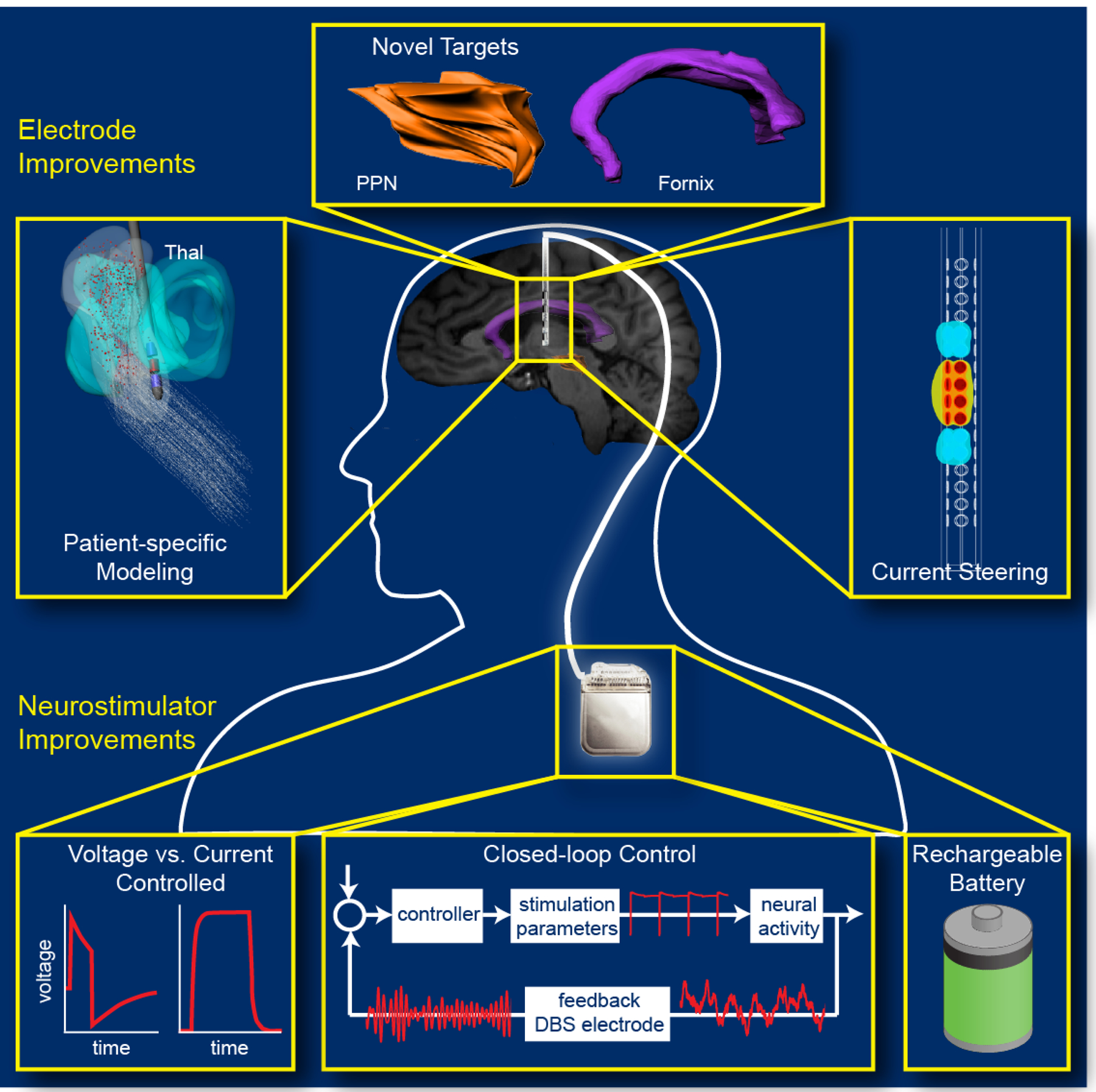
Deep brain stimulation for Parkinson’s disease [129]. The stimulation lead is implanted to a deep brain structure, and connected to the pulse generator in the chest via a lead tunneled through the neck (left panel). For Parkinson’s disease, the stimulation lead is targeted to either the internal segment of the globus pallidus (middle right panel) or to the sub-thalamic nucleus (lower right panel). Adapted from Ref. [129] with permission.

DBS was first introduced by Hassler et al. in the 1950s [126]. Current DBS systems require a lead of electrodes to be surgically implanted in the brain, typically in a deep brain structure within or near the thalamus, along with an implantable pulse generator (IPG) in the chest. Stimulation is typically delivered at a high frequency (60–185 Hz) from 0–10 V [127]. The effect of DBS depends on the physiological properties of the tissue, stimulation parameters, and electrode-tissue interface. One of the first clinical applications of DBS was for movement disorders, via stimulation of the thalamus [128], sub-thalamic nucleus, or globus pallidus, for reducing tremor, as shown in Figure 8 [129]. DBS has also been applied to epilepsy in order to reduce seizures through stimulation of the anterior nucleus of the thalamus or hippocampus. The applications of DBS have expanded to include chronic pain, dystonia, and obsessive-compulsive disorder, as well as other applications that are currently being explored [130].

Intracranial electrical cortical stimulation was introduced for modulating brain activity in 1954 [131]. Invasive cortical stimulation typically involves implanting an electrode array between the skull and cortical surface, and applying electrical stimulation via the electrodes using an IPG, similar to DBS [132]. Cortical stimulation devices often include an array of electrodes for the modulation of a larger patch of the cortex. The effects of cortical stimulation depend on the electrode polarity, stimulation parameters, and proximity to neuronal processes—especially dendritic structures. While epidural electrodes, placed over the dura mater, are the most common, subdural electrodes, placed directly on the cortex, are also available and may offer increased precision. In addition to surface electrodes, penetrating cortical electrodes have been developed, offering increased access to deeper layers of cortex, along with patient-specific cortical electrode designs [133]. The most prominent clinical application of cortical stimulation is epilepsy, as a means of aborting sensed epileptic activity via intracranial cortical electrodes and closed-loop control [134].

#### 4.1.2 Noninvasive neuromodulation

Many noninvasive approaches for neuromodulation have been developed to enable the modulation of neural tissue without necessitating invasive surgical procedures, including transcranial magnetic stimulation (TMS), transcranial current stimulation (TCS), and transcranial focused ultrasound stimulation (tFUS), as shown in Figure 7. Noninvasive approaches tend to suffer from lower spatial resolution compared with invasive approaches, but they also carry lower overall risk due to their noninvasive nature.

TMS uses the principle of electromagnetic induction to induce electrical currents within the brain [135, 136]. TMS was first introduced in 1985, and has since emerged as a neuromodulation therapy for a variety of disorders [137]. During TMS, current is pulsed through a coil of wire, creating a time-varying magnetic field that elicits eddy currents within the brain when transmitted through a nearby conductor. The resultant eddy currents can be strong enough to generate synchronous activity and action potentials. The TMS coils typically have a figure-eight configuration [138], ensuring current summation and thereby maximal activation at the adjoining edge of the two coils. Standard TMS coils have either 50 mm or 70 mm loops, with cortical activation areas as small as 1 cm^2^, depending on the stimulation parameters [135]. Alternative coil designs, such as the Hesed coil (H-coil) have also been developed to allow for the stimulation of deeper brain structures than those reached by traditional coils [139]. H-coils were designed to offer slower decay of the induced electric field over depth, allowing for stimulation of deeper brain structures, although with less focality than traditional coils [140, 141]. The modulatory effects of TMS are determined by the intensity, duration, and especially by the frequency of stimulation, with high-frequency stimulation (> 5 Hz) considered to be excitatory and low-frequency stimulation (< 1 Hz) considered to be inhibitory. The extent of modulation also depends on the underlying neuronal population, the direction of current flow relative to the neuronal population, waveform shape, and tissue conductivity. Applications of TMS include depression [142], for which it is approved by the Food and Drug Administration (FDA), stroke recovery, and Parkinson’s disease, among others [143].

TCS is a technique that has been used since ancient times and was recently reintroduced as a form of noninvasive brain stimulation for modulating cortical excitability [144]. The technique uses low levels of current applied to the scalp in order to modulate cortical activity. Transcranial direct-current stimulation (tDCS) uses weak, direct currents to elicit changes in cortical excitability and spontaneous neural activity, while transcranial alternating-current stimulation (tACS) uses currents with alternating polarities to similarly alter spontaneous activity and potentially entrain neural oscillations. TCS is generally considered a subthreshold stimulation technique, in that it modulates excitability without generating action potentials directly. Two large (5 by 7 cm) sponge electrodes, soaked with saline solution, are typically applied to the scalp, with 0.5–2 mA of current flowing from the negative cathode to the positive anode for 10–20 min. In general, anodal stimulation is considered to be excitatory and cathodal stimulation is considered to be inhibitory [145], and the activated cortical area is typically on the order of several square centimeters [135]. High-definition tDCS (HD-tDCS) electrodes (1 cm in diameter) have recently been introduced for increased focality and intensity compared with standard tDCS [146]. tDCS has been explored as a treatment for a variety of conditions, including depression, schizophrenia, and Parkinson’s disease.

tFUS has recently emerged as a neuromodulation technique. US has been widely used as a diagnostic imaging technology for years, and the notion of US activating neuronal tissues was first described in 1929 [147]. However, the concept of modulating ongoing brain activity using pulsed US without destroying the underlying tissue has only recently gained attention in the field of neuroscience. US neuromodulation uses low-intensity and low-frequency pulsed US waves that activate a non-specific population of cells within the acoustic pressure field [148]. Thermodynamic investigations have shown that tissue-heating does not occur within brief exposure times, but mechanical waves can propagate through neuronal membranes to influence the fluidity and excitability of ion channels and cell membranes. Although US has been explored widely in animal models, tFUS has only recently been demonstrated to modulate ongoing neural activity in human brain circuits [149, 150]. A recent experimental study has shown its potential application as a therapeutic method for brain injury [151]. Although focused US is in the early stages of human applications, theoretically it could offer enhanced spatial resolution and targeting relative to other noninvasive techniques, given the highly focused energy source that is delivered.

### 4.2 Current research and trends in neuromodulation

#### 4.2.1 High spatial and temporal specificity

Increasing the spatial and temporal resolution of all modalities is a prominent area of research in neuromodulation. With increased spatial resolution, the specificity of stimulation can be improved in order to ensure that the desired neuronal tissue is activated while avoiding stimulation of nearby areas, which could lead to unwanted side effects. For example, within the DBS field, advanced leads have been introduced with dense arrays of radially segmented electrodes to allow for increased selectivity and current steering around the lead [152] in order to maximally activate the targeted area while avoiding undesired side effects. Similarly, there is a push in the cortical stimulation field toward the use of microelectrodes and smaller surface electrodes for enhanced specificity and selectivity. In the noninvasive realm, research has focused on achieving more focal activation of cortical areas. For TMS, the current generated in the cortex is largely dependent on the coil design. Advanced coil designs and stimulation circuitry that allow for increased precision in cortical activation while avoiding coil overheating are being developed, including multichannel configurations and alternative coil designs [153, 154]. With respect to tDCS, high-definition electrodes have been developed that allow for more precise targeting of cortical regions [146, 155].

Integrating high-resolution neuroimaging with neuromodulation can also enhance spatial and temporal specificity. High-field MRI has been integrated into pre-surgical planning for DBS implantation, providing high-resolution images of thalamic nuclei to aid in DBS lead placement. Neuronavigation, using previously acquired structural or functional MRI data, is increasingly being used to guide the location and orientation of noninvasive neuromodulation technologies. The development of MR-compatible neuromodulation devices, especially with respect to DBS, is an important area of current research, in order to enable imaging after lead implantation to verify the lead location and the functional implications of the stimulation [156]. MR-compatibility in neuromodulation systems requires not only advanced hardware design and materials, but also careful selection of MR sequences to ensure subject safety when exposed to MRI radiofrequency pulses. Most commercially available devices are suitable for low field strengths (up to 1.5 T), but additional research is required to ensure compatibility at higher field strengths. If this technical challenge is overcome, high-field MR images could be used in real time to guide lead placement during surgical implantation, potentially improving patient outcomes.

#### 4.2.2 Closed-loop neuromodulation

Closed-loop stimulation is a promising research area that allows for responsive stimulation and real-time symptom management. Most available neuromodulation devices are open-loop, in which the physician closes the loop between the stimulation parameters and behavioral outcome. Especially in the invasive realm, the process of identifying ideal stimulation parameters in an open-loop fashion is time consuming, and may not result in the optimal parameters being selected. With increasing electrode numbers for implanted arrays, the stimulation parameter space has quickly expanded, making optimal stimulation even more tedious to identify. If neural sensing is incorporated into neuromodulation techniques, the stimulation could be automatically adjusted using control strategies in response to changes in the sensed signal. In Parkinson’s disease, for example, the power in the beta frequency band has been suggested as a potential control signal for closed-loop DBS therapy, as the signal is reduced during medication and DBS therapy [157]. Demand-driven stimulation has begun to be implemented in the field of epilepsy, with a closed-loop system currently under clinical investigation to sense and abort epileptic activity via intracranial cortical electrodes [134]. Implementing such closed-loop stimulation, however, would require a robust biomarker across patients, as well as advanced IPG hardware to compensate for the additional computational power that would be required. In the noninvasive realm, closed-loop tDCS and EEG have begun to be explored for individualized cognitive training and rehabilitation, as well as for epilepsy management. Regardless of the application, advanced control strategies will be required to further characterize the ideal transfer functions for each modality and application.

#### 4.2.3 Neuromodulation for perturbation-based imaging

Although most neuromodulation is used therapeutically, it can also be used to perturb the nervous system in a controlled way. This perturb-and-record method has been used extensively in clinical applications in order to delineate eloquent areas during surgical resection. However, such perturbation-based imaging can also be used to gain information about the functional consequences of various brain areas—with high spatial and temporal precision [13]. When used in this way, neuromodulation allows for the temporary alteration of brain networks, while neuroimaging techniques can be used to assess the functional implications of such alterations. Perturbation also allows for the distinction between correlation and causation, by tracking the propagation of induced activity through a network of brain areas in space and time. In this way, perturbation-based imaging could serve as a complement to traditional anatomical and functional mapping, and could allow for the direct testing of hypotheses regarding brain function that would otherwise be inaccessible.

#### 4.2.4 Cell-type specificity in stimulation

Optogenetics is an emerging technology that allows for the controlled activation of selected and specific cell types, adding immense potential for cell-type specificity to neuromodulation [68]. The principle of optogenetics involves the viral transfection of rhodopsin genes, causing cells to express channel rhodopsin proteins, which react to specific wavelengths of light to depolarize or hyperpolarize the cell [158]. This cell-specific approach clearly offers superior spatial specificity relative to other techniques [159]. While typically invasive, noninvasive optogenetic approaches have recently been demonstrated in animals [160]. Advanced optical fiber probes are also being developed to allow for simultaneous stimulation and fluorescence detection [161]. The combination of optogenetics with neuromodulation modalities would allow for the addition of cell-type specificity to current stimulation methods, enabling unprecedented spatial resolution and selectivity. However, the integration of optogenetics with neuromodulation technologies poses several challenges, and the functional resolution is limited by the location and density of protein expression. In addition, the requirement for genetic manipulation, on a permanent basis, limits the application of optogenetics to animal models for the foreseeable future, and its use in human tissue poses significant scientific and ethical challenges.

### 4.3 Challenges and future directions

Although the field of neuromodulation holds great promise for advancing our understanding of the brain and for the treatment of neurological disorders, several challenges remain in advancing the field beyond its present state. The therapeutic effects of neuromodulation technologies require optimization to account for variability among conditions and individuals. Robust biomarkers need to be identified, both of disease states and of therapy, in order to better understand the mechanisms underlying therapeutic stimulation. The demonstration of long-term efficacy in some applications, such as Parkinson’s disease, has highlighted the potential gain of using neuromodulation in other unexplored areas, such as affective disorders [162]. To expand the applications into novel conditions and to further understand the mechanisms of stimulation, robust translational and computational models will be needed in order to inform and design therapies. The addition of other techniques, such as optogenetics, could also enable enhanced understanding of the cellular mechanisms associated with therapeutic neuromodulation, as well as a narrowing of the present disconnect between stimulation parameters and behavioral effects. The therapeutic variability among individuals and conditions also merits further attention. With respect to individual variation, genetic factors such as brain-derived neurotrophic factor have been shown to be correlated with differences in efficacy between responders and non-responders, especially in the noninvasive realm [163]. Across conditions, the time-course of therapeutic effects varies drastically for identical neuromodulation technology. For example, DBS treatment for Parkinson’s disease can immediately reduce tremors, while DBS treatment for dystonia can take weeks or months of continued stimulation to achieve therapeutic effects [162]. The fundamental cause of such time-course differences remains poorly understood, but will be crucial to the development and optimization of future neuromodulation technologies. Future neuromodulation approaches will very likely need advanced stimulation technology with optimal spatial and temporal precision. Devices with increased degrees of freedom for stimulation will be needed in order to achieve enhanced selectivity to ensure that the optimal tissue is activated. Future research will need to elucidate the optimal frequency, duration, and intensity of stimulation for a variety of technologies and diseases. In addition, the ideal relationship between neuromodulation and other therapeutic approaches, such as behavioral therapy or medication, requires further exploration, as combinatorial therapies may offer greater gains than either treatment alone. Further development of subject-specific targeting, via computational models and navigation with imaging, will also be important in order to delineate optimal targets and enhance therapeutic outcomes. Lastly, the tissue-electrode interface will require improvement to ensure that neuromodulation technologies offer longevity in treatment efficacy and safety.

## 5 Conclusion

In this paper, the basic concepts, trends, and challenges in the field of systems neuroengineering, including neuroimaging, neural interfacing, and neuromodulation, were reviewed with the intent of demonstrating how these fields play a key role in the process of designing and developing neuro-devices. These neurotechnologies play an important role in tackling grand challenges in interfacing the engineering and physical sciences with life sciences and medicine [13]. As mentioned previously, in order to understand the organization of neural systems, neuroimaging is essential. Obtaining knowledge of the underlying brain processes and information flow within various brain regions is critical for understanding the brain. This understanding can be used to enhance neural communication in dysfunctional cases by developing neural interfaces and BMI technologies, or to treat pathological activity via neuromodulation techniques.

As neural processes within the brain are realized through highly specific, distributed, and dynamic networks, it is essential that neuro-devices are capable of recording with high spatiotemporal resolution [13]. These devices will need to decode various brain states in order to determine the neural behavior characterizing both desired and undesired activity. Optimal devices will be those that can then interact with targeted brain circuits to achieve the preferred effect, while minimizing undesirable side effects that may result from activating extraneous brain networks. Therefore, the future generation of neuro-devices will require increased specificity, perhaps through multimodal integrative approaches, in order to achieve such selectivity.

While neuro-devices are typically perceived as effectors through the lens of neuromodulation, they can also be used to study underlying brain networks and processes by implementing perturbation-based imaging methods. In this manner, neuro-devices with high spatiotemporal resolution can perturb specific networks and subsequently record the brain’s response. These techniques can elucidate the roles of various brain regions and structures in network activity. This knowledge may aid in classifying disease versus healthy brain states and may help in the development of therapeutic targets for treatment options. The design and development of future neuro-devices—whether for modulation, studying the brain, or both these applications—will require the intersection of advanced research in neuroimaging, neural interfacing, and neuromodulation. Such a systems-level approach toward neuroengineering will be crucial for advancing our understanding of normal and pathological states of the brain.
